# Hybrid Therapy with SBRT Target-Tailored Tumor Resection for High-Grade Metastatic Epidural Spinal Cord Compression (MESCC): Illustrative Case

**DOI:** 10.3390/jcm14051688

**Published:** 2025-03-03

**Authors:** Mario De Robertis, Lorenzo Lo Faro, Linda Bianchini, Ali Baram, Leonardo Anselmi, Elena Clerici, Pierina Navarria, Marco Riva, Marta Scorsetti, Federico Pessina, Carlo Brembilla

**Affiliations:** 1 Department of Biomedical Sciences, Humanitas University, Via Rita Levi Montalcini 4, 20090 Pieve Emanuele, Milan, Italyfederico.pessina@hunimed.eu (F.P.); 2 Department of Neurosurgery, IRCCS Humanitas Research Hospital, Via Manzoni 56, 20089 Rozzano, Milan, Italycarlo.brembilla@humanitas.it (C.B.); 3 Department of Radiotherapy and Radiosurgery, IRCCS Humanitas Research Hospital, Via Manzoni 56, 20089 Rozzano, Milan, Italyelena.clerici@humanitas.it (E.C.);; 4 Independent Researcher, 27100 Pavia, Pavia, Italy

**Keywords:** metastatic epidural spinal cord compression, Hybrid Therapy, Separation Surgery, SBRT, neuronavigation

## Abstract

**Background:** Spinal metastases affect approximately 40% of patients with systemic cancers; metastatic epidural spinal cord compression (MESCC) occurs in up to 20% of cases and leads to potential significant morbidity. Recent advancements in high-dose conformal radiation techniques, such as Stereotactic Body Radiation Therapy (SBRT) and Stereotactic Radiosurgery (SRS), enable histology-independent ablative treatments, yet optimal dose fractionation remains undetermined. **Methods and Results:** This case of vertebral metastases with high-grade ESCC exemplifies the model of a comprehensive treatment workflow that emphasizes interdisciplinary collaboration, within the framework of a personalized medicine. The “Hybrid Therapy” combines Separation Surgery, aimed at achieving circumferential spinal cord decompression, with SBRT/SRS. The oncologic resection has been performed in a navigation-assisted technique that is tailored to the SBRT target, pre-operatively defined on the neuronavigation station. **Conclusions:** This seamless integration during initial planning of surgery with the ideal radio-oncological target is aimed at avoiding delays in referral and limitations in subsequent treatment options. This integrative holistic strategy not only prioritizes functional preservation, minimizing surgical invasiveness, but also promotes tumor control, thus offering potential promising new avenues for patient-centered oncologic care. Future high-quality studies are warranted to validate the widespread potential utility and safety of this approach.

## 1. Introduction

Spinal metastases impact approximately 40% of individuals diagnosed with systemic cancers. The overall incidence of spinal metastases is expected to increase due to advancements in radiotherapy technologies and the spread of target therapies: both are contributing positively to local control and survival rates of patients with various forms of systemic cancer [1,2,3].

Metastatic epidural spinal cord compression (MESCC) occurs in up to 20% of cases and is an increasingly prevalent clinical condition among cancer patients, associated with significant morbidity and neurological sequelae. Pain is the most prevalent symptom in patients with metastatic spinal disease, occurring in 80–95% of cases, followed by motor radiculopathy. However, it represents the initial presenting symptom in only approximately 10% of patients [4]. In these cases, pain may precede the onset of neurological deficits by weeks or even months [5,6]. The etiology of pain in spinal metastases can be attributed to one or a combination of three mechanisms: local pain, mechanical pain, and radicular pain. Bladder and bowel symptoms are present in more than half of the patients with spinal cord compression at the time of diagnosis [7].

It is suggested that up to 50% of diagnosed spinal metastases require some form of therapeutic intervention, with 5–10% necessitating surgical management. Historically, treatment has relied on a multidisciplinary strategy incorporating surgery, radiation therapy, and systemic treatments, each customized to the patient’s tumor burden, overall health, and prognosis. The management of spinal metastases is primarily palliative, with the main objective being the preservation or enhancement of the patient’s quality of life. This goal encompasses effective pain control, mitigation of neurological deficits, and restoration of ambulatory function [7,8]. Given the heterogeneous presentation and variable treatment responses of spinal metastases, a multidisciplinary tailored approach is essential for optimal patient outcomes.

Over the past fifty years, MESCC management has undergone several substantial paradigm shifts. With the advent of more effective systemic therapies extending patient survival, there is increasing interest in achieving durable local control (LC) and preventing neurological compromise in selected patients. Simultaneously, advances in conformal radiation delivery technologies, particularly through Stereotactic Body Radiation Therapy/Stereotactic Radiosurgery (SBRT/SRS), have enabled the safer application of photon beam therapy, resulting in histology-independent ablative effects, further broadening the spectrum of available therapeutic options [9,10]. The precise delivery of high biologically effective doses (BEDs) of radiotherapy via SBRT to the spine has been shown to provide sustained local tumor control and significant pain relief. In patients with oligometastatic disease, SBRT targeting known metastatic sites can prolong progression-free survival and potentially delay the need for subsequent lines of systemic therapy. In the postoperative setting, SBRT contributes to maintaining neurological function by enhancing local disease control. Moreover, for patients with prior spinal irradiation, SBRT offers a safe retreatment strategy for the same or adjacent vertebral segments while minimizing radiation exposure to critical neurological structures [11,12]. However, the optimal dose schedule (single-fraction versus hypo-fractionation), the ideal target coverage, and the prescription dose have not yet been established. Future rigorous randomized clinical studies comparing dose fractionation regimens will be crucial, as dose-escalation has shown promise for enhancing local control [13,14]. Some authors suggest that the severity and onset of neurological symptoms are particularly relevant in a subset of patients with good performance status who have had minimal exposure to systemic treatments that could potentially extend survival. Neurological assessment, incorporating both clinical evaluation and imaging-based scoring (Bilsky Score [15]), alongside considerations of spinal stability and tumor radio-sensitivity, may guide the most appropriate treatment approach. Patchell et al. advocate for surgical decompression followed by conventional radiotherapy as the first-line treatment for symptomatic single-level ESCC, supported by level one evidence. They utilized ambulation as an indicator of local control, reporting higher ambulatory rates of 84% following surgical decompression combined with conventional EBRT, compared to 57% for EBRT alone. However, local failure rates were high at 69.3% and 96% at 1 and 4 years, respectively [16]. Similarly, Rothrock et al. report improved overall survival in patients undergoing surgery for spinal metastases, highlighting the growing role of adjuvant therapy in enhancing survival and quality of life [17]. Previous studies indicate that EBRT (such as 20 Gy in 5 fractions or 30 Gy in 10 fractions) is widely utilized for spinal metastases, providing effective pain relief, particularly in radiosensitive tumors. However, local control remains variable, and the need for multiple fractions increases the risk of complications such as pseudoarthrosis. Data interpretation, concerning SBRT and EBRT comparison, is challenging, as many studies lack direct imaging-based assessment of local control during follow-up [18]. A randomized phase II trial compared upfront (i.e., primary) SBRT and EBRT, focusing on pain relief at three months as the primary endpoint [19]. Utilizing a visual analog scale, the study found that SBRT provided superior pain relief at three months, achieved more rapid alleviation of pain, and maintained better pain control at six months compared to EBRT [20]. However, regardless of the regimen adopted, SBRT delivers substantially higher BEDs compared with conventional EBRT, with positive impact on local control [21]. It is important to emphasize that neither radiotherapy technique nor fractionation regimen can address spinal instability or fractures that require a surgical strategy. Furthermore, there is currently a lack of reliable comparative data to determine the optimal rates of re-calcification and pathological fracture prevention across different radiotherapy regimens, as these outcomes may depend on tumor histology and concurrent systemic therapies (e.g., bone-modifying agents) [18].

Nonetheless, despite the several advancements, significant disparities persist in the global management of spinal metastases. This variability underscores the need for standardized treatment protocols, driving the development of consensus guidelines informed by current evidence and expert consensus to support clinical decision-making.

In this context, the progressive adoption of Hybrid Therapy (HT)—the combination of circumferential neural decompression (Separation Surgery—SS) and adjuvant SBRT/SRS—has facilitated less aggressive surgical approaches, limiting the extent of resection while optimizing the radio-oncological target and reducing the risk of overdose to adjacent critical structures [22]. The Separation Surgery technique incorporates some standardized maneuvers that allow for dural sac decompression via a posterior transpedicular approach. However, excessive unintentional debulking may compromise the stability of the anterior column, necessitating anterior reconstruction, prolonging surgical duration and exposing the patient to unnecessary morbidity in terms of functional outcomes, local control, and survival.

In the era of precision medicine, the pursuit of less or minimally invasive strategies is of paramount importance. Navigation-assisted oncologic spine surgery enhances precision in instrumentation placement and serves as a valuable tool for guiding tumor resection and debulking. This approach reduces morbidity, improves real-time anatomical visualization, and streamlines the treatment workflow from preoperative planning to intraoperative decision-making. Moreover, significant advancements in surgical technology, particularly in Minimally Invasive Surgery (MIS) techniques, contribute to improved patient outcomes and accelerated recovery [23]. Additionally, the seamless integration of radiotherapy with surgery—particularly in cases of metastatic tumors with high-grade neural compression—plays a crucial role in oncologic patient management and helps prevent treatment delays.

Here, we present the proposal of a workflow model for a navigation-assisted oncologic resection technique, tailored to the preoperatively defined ideal radiotherapy target, for patients with high-grade epidural compression eligible for Hybrid Therapy.

## 2. Materials and Methods

### 2.1. History and Presentation

A 70-year-old female patient presented with progressively worsening mechanical axial pain persisting for nearly three weeks prior to her emergency room access. Her medical history included hypertension, type 2 diabetes mellitus, dyslipidemia, postmenopausal osteopenia, vitamin D deficiency, stage C heart failure with preserved left ventricular systolic function, and Hashimoto’s thyroiditis.

**Figure 1:**
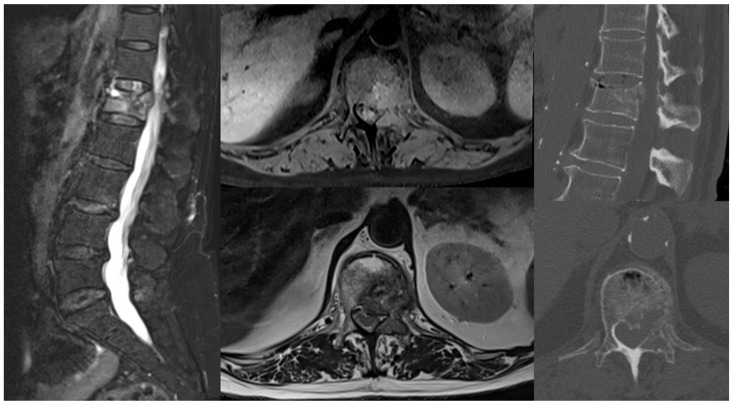
Pre-operative MRI and CT assessment. L1 pathologic fracture with epidural spinal cord compression of Bilsky grade 2.

Nine years earlier, she had undergone a right quadrantectomy followed by adjuvant radiotherapy for luminal A breast cancer and therapy with anastrozole for five years. Five years later, due to a shift to a triple-negative histotype, she received a total mastectomy and subsequent chemotherapy with cyclophosphamide, methotrexate, and fluorouracil. At her last follow-up, five months prior to presentation, she was disease-free. Upon admission to the Neurosurgery Department, she was neurologically intact (ASIA E) but experienced stabbing back pain (NRS 9/10), particularly during ambulation, and L1 radicular pricking pain (NRS 6/10). EQ-5D VAS was used as a simple tool for general Quality-of-Life measures at baseline and during follow-up: the baseline score was 40. A total-body CT scan and lumbar MRI with contrast were conducted for restaging, revealing a pathological fracture at L1 with grade 2 anterolateral left epidural cord compression, epiconus displacement, and a lesion in the left acetabulum (Figure 1). The lesions appeared lytic on CT imaging, and the Spinal Instability Neoplastic Score (SINS) was 11.

### 2.2. Pre-Operative Planning and Treatment Strategy

Given the patient’s oligometastatic profile and favorable performance status (ECOG 1), a multidisciplinary team recommended Separation Surgery followed by adjuvant stereotactic body radiation therapy (SBRT).

The day before surgery, CT and MRI images were uploaded to the StealthStation S8 Medtronic system under a dedicated profile labeled “Hybrid Therapy” within the cranial section. A radiation oncologist, in collaboration with a medical physicist, developed the treatment plan directly on the workstation. Following image fusion, the plan was created based on standard criteria for the proper adjuvant radiation therapy. The target lesion was delineated with a red line, allowing for pre-operative evaluation of its three-dimensional reconstruction and volume within the vertebral segments.

**Figure 2:**
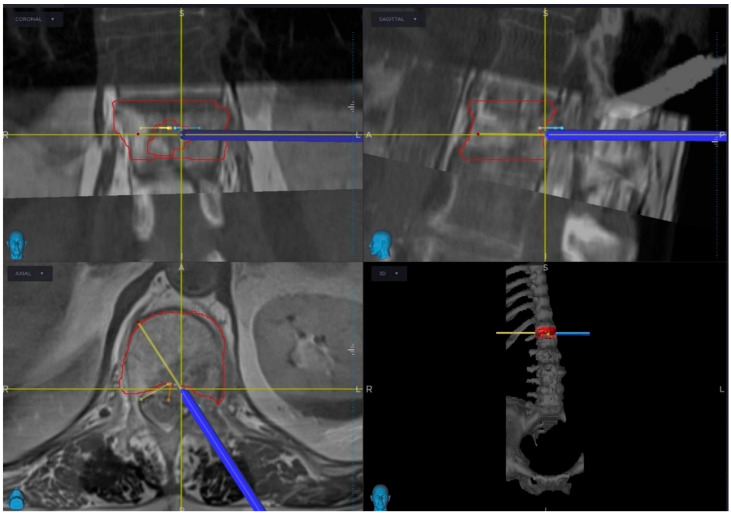
Intraoperative manual imaging merging of pre-operative standard lumbar MRI with the registered SBRT target plan and 3D-CT scan acquired with O-Arm Medtronic, after positioning of the D12 spinous passive frame. The pointer identifies the left trans-pedicular resection trajectory to perform Separation Surgery.

The patient was positioned prone, and a linear midline incision was made, centered at D12-L2. After exposing the vertebral posterior arches, a passive frame was positioned at the D12 spinous process. A 3D-CT scan with O-Arm was performed intraoperatively and interfaced with the Medtronic neuro-navigation system. The preoperative plan was manually fused and verified against the acquired images (Figure 2). Carbon fiber-PEEK (Poly-ether-ether-ketone) hardware was selected to enhance surveillance imaging quality, optimize radiation target coverage, and minimize exposure to organs at risk (OARs). Bilateral pedicle screws were placed in D12 and L2. The ability to use only the pointer in the cranial section of the navigation software precluded the possibility for a navigating trocar aimed at a percutaneous approach; thus, we decided to perform an open short-segment fixation with screw augmentation, thereby reducing muscle dissection and minimizing blood loss to enable early access to adjuvant radiotherapy. A second 3D-CT scan was obtained intraoperatively to confirm accurate screw positioning and PMMA (Poly-methyl-methacrylate) distribution.

**Figure 3:**
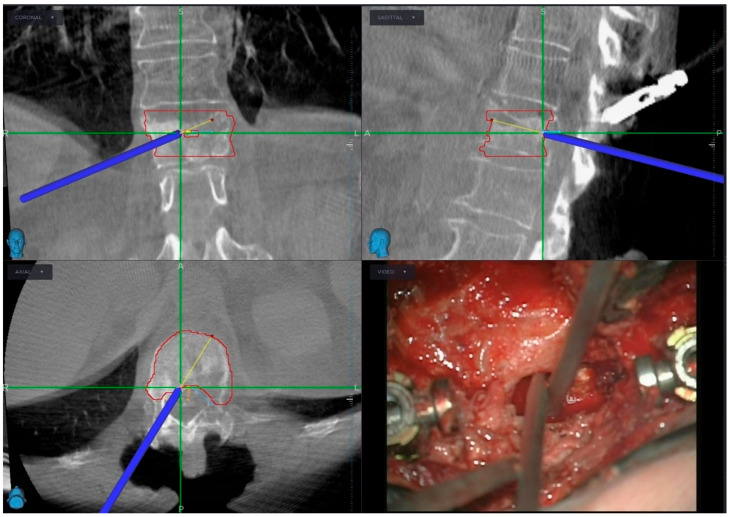
Controlateral (right) trans-pedicular approach to decompress ventrally the dural sac, following the SBRT-target boundaries.

**Figure 4:**
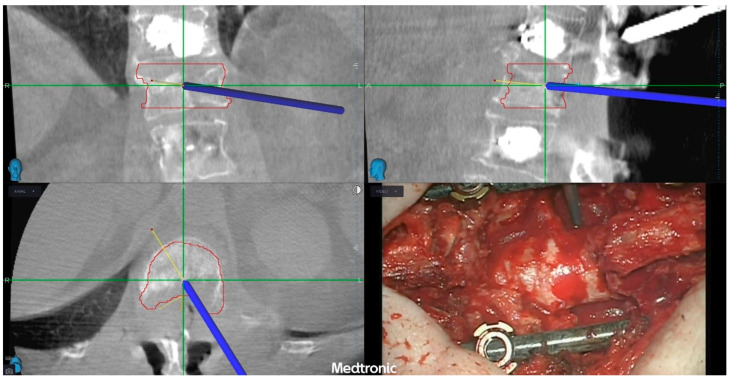
Final check after rod placement: given the potential for tumor regrowth and the evident manual merging error—partly due to the absence of an adequate volumetric lumbar CT/MRI imaging—the resection margin extends deliberately slightly beyond the minimum SBRT-target boundary.

A high-speed drill was used to perform a laminectomy at L1 following tulip distractor placement. Bilateral pediculectomy was then conducted, and circumferential decompression of the lesion was achieved in a stepwise manner with navigation assistance. The resection, integrated with the microscope view, was extended deliberately slightly beyond the red target line to account for a potential tumor regrowth until SBRT (Figure 3). No nerve roots were sacrificed. The posterior longitudinal ligament was dissected anteriorly from the dural sac and resected. Upon completion, the spinal cord was fully decompressed as confirmed by the navigation check (Figure 4). Finally, carbon fiber-PEEK rods were placed.

### 2.3. Radiotherapy Planning and Delivery

Four weeks postoperatively, the patient underwent the SBRT simulation procedures. As for the simulation CT scan, the patient was positioned supine and immobilized with a thermoplastic body mask that covered the abdominal and pelvic region. CT was acquired with 1 mm thick slices, and no mean of contrast was needed. Later on the same day, simulation dorsal-lumbar MR with gadolinium was taken, and images were fused for contouring purposes. The Gross Tumor Volume (GTV) corresponded to the residual disease after surgery identified at the post-operative imaging; the Clinical Target Volume (CTV) was contoured encompassing both the initial lesion at the pre-operative imaging and the GTV; then, a 1 mm isotropic expansion to Planning Target Volume (PTV) was generated. The adjacent organs at risk (OARs) were the following: left and right kidney, small bowel, liver, and spinal cord; for the latest, a cautionary 1 mm expansion to spinal cord Planning Organ at Risk Volume (PRV) was performed. Dose constraints and treatment specifics are illustrated in Table 1.

The total dose used was 24 Gy in two fractions prescribed homogeneously, trying to avoid hot spots, especially around the spinal cord. Treatment was delivered on a Varian EDGE linear accelerator using a 10 MV Flattening Filter Free (FFF) photon beam with the Volumetric Modulated RapidArc technique (VMAT-RA). Three-dimensional image-guided radiation therapy (IGRT) by cone beam CT (CBCT) was performed before each daily session to assess the correct patient and tumor positioning.

Radiotherapy toxicities were evaluated following the Common Terminology Criteria for Adverse Events (CTCAEs) score v. 5.0. Treatment was well-tolerated in the acute setting, with the patient experiencing only mild fatigue and a minimal and temporary increase in lumbar pain, both events classified as G1 according to CTCAE.

**Figure 5:**
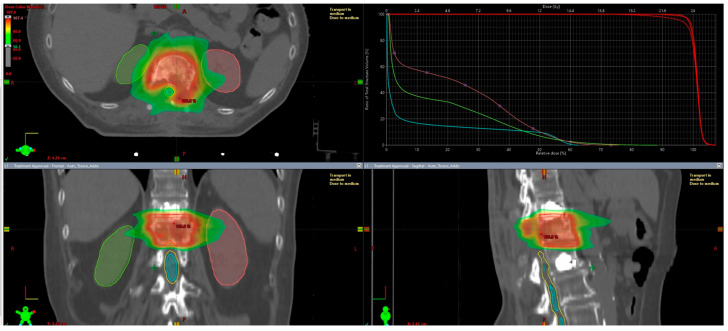
SBRT plan. Dose constraints: single kidney: Dmax ≤ 26 Gy, Dmean ≤ 6 Gy. Liver: reduction in Dmean as reasonably as possible. Small bowel: Dmax ≤ 20 Gy. Spinal Cord PRV: D0.035 cc ≤ 17 Gy (mandatory) and Dmax ≤ 19.3 Gy (mandatory); Dmax ≤ 17 Gy (optimal).

The SBRT plan is shown in Figure 5.

### 2.4. Ethics

The patient provided informed consent for both radio-surgical and research purposes.

### 2.5. Post-Operative Course and Outcome

The patient exhibited a progressive reduction in back pain, allowing for a rapid tapering of opioid medications. No intraoperative or postoperative adverse events were observed. On the first postoperative day (POD), active mobilization was initiated, and the urinary catheter and drainage were removed. A postoperative CT scan was conducted on the second POD. The patient was discharged on the seventh POD. Stereotactic body radiation therapy (SBRT) was well tolerated, with no complications reported.

At the 1-year clinical and radiological follow-up, there was no evidence of recurrence. MRI was required for tumor surveillance at 3, 6, and 12 months. At the last follow-up, the patient maintained an ECOG performance status of 0, with optimal decrease in mechanical axial pain (NRS 2/10) and disappearance of radicular pain. The EQ-5D VAS score was 90.

## 3. Discussion

The systematic evaluation of spinal metastatic disease prior to selecting an appropriate treatment modality is of paramount importance. This evaluation requires a comprehensive consideration of various factors, including the patient’s age, clinical symptoms, tumor histology, surgical tolerance, overall physical status, and anticipated survival outcomes. In this context, with the advent of SBRT/SRS, the NOMS (Neurologic, Oncologic, Mechanical, and Systemic) evaluation system has been adopted as a contemporary basic framework for assessing and making treatment decisions for spinal metastatic tumors [24].

SBRT/SRS offers significant advantages over traditional External Beam Radiation Therapy (EBRT). These advantages arise from the integration of advanced imaging technologies, sophisticated treatment planning software, image-guided positioning, and intensity-modulated dose delivery. Such innovations enable the precise delivery of high-dose radiation to the target area while minimizing exposure to adjacent normal tissues. This is particularly crucial in protecting sensitive structures, such as the spinal cord and cauda equina, thereby enhancing pain management and alleviating potential neurological symptoms, with myelopathy being one of the most severe SBRT complications [9,12,25]. The efficacy of SBRT/SRS is underscored by its histology-agnostic local control rates, which can exceed 86–90% across various tumor types; furthermore, hybrid treatment approaches that combine Separation Surgery with SBRT/SRS have demonstrated reduced surgical morbidity and improved long-term tumor control, decreasing the incidence of local recurrences [9,12,25,26]. Laufer et al. conducted a pivotal study involving 186 patients with high-grade ESCC, predominantly characterized by radioresistant tumor histologies, 50% of whom had previously failed radiation therapy. The observed one-year cumulative recurrence rate was 16.4%, with notably lower local recurrence rates of 9% and 4% for patients receiving the high-dose single-fraction and high-dose hypo-fractionated treatments, respectively [22]. A study by Moulding et al. showed LC rates of 91% at 1 year following post-operative SBRT to highly radioresistant histologies; this suggests that high-dose single-fraction SBRT can overcome radioresistance to conventional EBRT [27]. The findings from the multi-institutional randomized controlled SC24 trial further substantiate the superiority of SBRT in achieving pain control at three and six months post-treatment compared to conventional radiation therapy for painful spinal metastases [26]. In a recent systematic review, Wong et al. compared 3 randomized control trials comparing SBRT and conventional EBRT in the management of painful spinal metastatic lesions; 642 patients were included. A significant difference in complete pain response between SBRT and CBRT ranged from 33.3–35.1% and 13.1–13.9%, respectively, at 3 months [28]. Fuks et al. suggest that SBRT operates through the activation of alternative cell death pathways, such as the sphingomyelinase pathway; this pathway helps overcome hypoxia-induced resistance, which arises when tissues become devascularized in the post-operative setting [29]. An important role of SBRT concerns re-irradiation, which may involve irradiating the entire circumference of the spinal cord, presenting challenges due to the cumulative radiation dose. Therefore, careful planning is essential to minimize the risk of toxicity. A systematic review of nine studies investigating re-irradiation with SBRT reported a median one-year local control (LC) rate of 76%, with a range of 66–90%. The analysis also demonstrated an improvement in pain scores following re-irradiation, while treatment was considered safe, with a crude rate of vertebral compression fractures (VCFs) at 12% and radiation-induced myelopathy at 1.2% [30].

Concerning SBRT toxicity, acute side effects are frequently linked to the proximity of anatomical structures within the radiation field. For example, cervicothoracic SBRT may result in esophagitis, lumbar SBRT can lead to nausea, and sacral SBRT may cause loose stools. Additionally, acute pain is a common early side effect of SBRT, regardless of the treatment site. This typically develops within the first 24–48 h following SBRT and is observed in approximately 25% of cases [31]. VCF may develop either acutely or chronically. The risk of VCF was found to range between 10% and 40%, depending on the radiation dose and fractionation schedule. The most significant predictor of VCF was radiation dose, with the highest risk observed in fractions exceeding 20 Gy. Additionally, an association was identified with lytic lesions, spinal deformity, and baseline VCF. These findings suggest that caution is warranted when treating patients with fractures using doses greater than 20 Gy [32]. Supporting these results, a systematic review of 11 studies analyzing risk factors for VCF reported a crude VCF rate of 13.9% over a median follow-up period of 1.6–3.3 months [33]. Regarding post-operative complications, one study examining patients managed with surgery, with or without conventional EBRT or SBRT, reported a complication rate of 35%, including an 11.6% incidence of wound dehiscence [34]. These findings were comparable to another series in which all patients underwent post-operative EBRT or SBRT, reporting a complication rate of 30% and a wound infection rate of 10% [19]. A notable limitation of a combined approach (surgery + radiotherapy) is the potential for wound complications associated with early post-operative radiation treatment. To minimize the surgical footprint, the application of image-guided highly conformal radiation therapy may help preserve the operative corridor, thereby contributing to a reduction in postoperative complication rates [35]. Remond et al. suggested that SBRT may reduce radiation-related post-operative complications due to its conformal dose distribution, which enables selective wound sparing. Additionally, it has been hypothesized that SBRT could decrease the need for reoperation by reducing hardware failure, as not all implanted hardware is exposed to radiation [36]. However, despite SBRT’s potential for hardware sparing, the increased incidence of bone radionecrosis from higher per-fraction doses may result in a comparable failure rate within the more limited irradiated regions [21].

Moreover, Minimally Invasive Surgery (MIS) techniques like percutaneous approaches or tubular surgery have been increasingly practiced for spinal metastases and have been shown to decrease operative morbidity, promote a faster recovery, and enable safer access to adjuvant therapies [36]. Building on the promising preliminary results, high-quality studies with adequate levels of evidence are needed Another important consideration involves the choice of hardware materials. CFR-PEEK implants offer an elasticity modulus closer to that of bone, compared to metal implants, which reduces stress concentration at the bone–implant interface and enhances healing potential. Additional biomechanical advantages include lower wear rates relative to comparable materials, favorable mean bending yield load, bending ultimate load, and cycling capacity, and stiffness nowadays comparable to commercially available titanium systems. Moreover, CFR-PEEK has the great advantage of enabling superior-quality surveillance imaging. This plays a crucial role in adequate radiological follow-up definition and early and safer recurrence/regrowth detection, and makes radiotherapy plans easier by avoiding metal-related artifacts [37,38,39]. The support of precision medicine techniques in spinal surgery like neuronavigation technologies demonstrates promise in enhancing accuracy, safety, and patient outcomes. The identification of anatomical risks and the emphasis on preventive measures provide valuable insights for surgeons [23].

Patients with good performance status, oligometastatic background, and longer life expectancy at baseline prognostic assessment can benefit from Hybrid Therapy in the presence of high-grade metastatic epidural spinal cord compression. To the best of our knowledge, this constitutes the first report about the multidisciplinary integration of pre-operative radiotherapy planning with intra-operative real-time imaging technology aimed at defining and guiding the resection of spinal tumors. Understanding the technological trends can guide healthcare decision-makers in optimizing and innovating resource utilization.

### Limitations

**This is an illustrative case.** Its inherent nature, combined with the lack of statistical validation, limits the generalizability of the results. Indeed, the need for larger, high-quality studies is pivotal, underscoring the importance of robust research designs in establishing the widespread effectiveness and safety of this approach in spinal surgery.

**Cost of navigation and intraoperative imaging technology**. The implementation of advanced navigation systems and intraoperative imaging modalities, such as real-time 3D imaging and surgical navigation tools, remains a significant financial barrier, limiting their widespread adoption.

**Limited availability and reproducibility across centers**. The reproducibility of results when utilizing these advanced technologies can vary, as it depends on factors such as operator expertise, institutional resources, and equipment calibration, which may not be consistent across different settings. On the other hand, the use of a real-time surgical visualization system, specifically tailored to adjuvant therapy, can also assist a surgeon who is less confident with the surgical technique of Separation Surgery.

**Absence of preoperative volumetric imaging and potential errors in manual merging**. Intraoperative imaging may not always align perfectly with preoperative planning; the manual fusion of preoperative and intraoperative images is highly operator-dependent and could introduce significant errors not currently quantifiable. The call for larger, high-quality multicentric studies to verify the utility and safety of this workflow model is of paramount importance, also to assess, as quantitatively as possible, the margin of error to be considered with a similar approach aimed, as mentioned, at further minimizing the extent of tumor resection. The fusion of non-volumetric CT/MRI with intraoperative O-arm 3D imaging can limit strict adherence to the defined target. Moreover, the possibility of merging pre- and post-operative imaging only in the “cranial” section of the Medtronic StealthStation S8 prevents the performance of a percutaneous navigation-assisted approach. This represents a significant limitation, considering the growing availability of minimally invasive surgery (MIS) techniques. However, this could provide the basis for the creation or optimization of dedicated software aimed at addressing these limitations, with the obvious necessity of testing it on a large scale.

## 4. Conclusions

The present case underscores the importance of a comprehensive treatment workflow encompassing every step from preoperative planning to intraoperative decision-making. Our approach emphasizes close interdisciplinary collaboration, facilitating upfront seamless integration of SBRT, to avoid delays in referral and limitations in subsequent treatment options. The method provides guidance in determining the optimal transition point between surgical resection and SBRT, ensuring that decisions are made with a holistic view of patient risks and treatment benefits. This integrative strategy not only prioritizes functional preservation but also promotes tumor control, offering potentially promising new avenues for patient-centered oncologic care.

## Figures and Tables

**Table 1:** Dose constraints and Treatment specifics.

**ORGAN**	**Constraints to Be Respected**	**Treatment Specifics**
**PRV Spinal Cord**	D0.035 cc ≤ 17 Gy (mandatory) Dmax: ≤17 Gy (optimal) ≤19.3 Gy (mandatory)	D0.035 cc = 16.47 Gy Dmax = 18.31 Gy
**Small Bowel**	Dmax ≤ 20 Gy	Dmax = 12.03 Gy
**Single Kidney**	Dmax ≤ 26 Gy Dmean ≤ 6 Gy	Dmax (left) = 18.2 Gy; Dmax (right) = 21.19 Gy Dmean (left) = 5.39 Gy; Dmean (right) = 3.64 Gy
**Liver**	Dmean as low as reasonably possible	Dmean = 1.19 Gy
**Target Coverage**	V_95%_ > 95%	V_95%_ = 99.6%

## Data Availability

The original contributions presented in this study are included in the article. Further inquiries can be directed to the corresponding author.

## Abbreviations

The following abbreviations are used in this manuscript:

MESCC	Metastatic Epidural Spinal Cord Compression
SBRT/SRS	Stereotactic Body Radiation Therapy/Stereotactic Radiosurgery
EBRT	External Beam Radiation Therapy
LC	Local Control
BED	Biological Effective Dose
HT	Hybrid Therapy
SS	Separation Surgery
MIS	Minimal Invasive Surgery
PMMA	Poly-methyl-methacrylate
PEEK	Poly-ether-ether-ketone
CTV	Clinical Target Volume
PTV	Planning Target Volume
OAR	Organs at Risk
PRV	Planning Organ at Risk Volume
FFF	Flattening Filter Free
VMAT-RA	Volumetric Modulated RapidArc technique
IGRT	Image-Guided Radiation Therapy
CB-CT	Cone Beam CT
VCF	Vertebral Compression Fracture
CTCAE	Common Terminology Criteria for Adverse Events
POD	Post-Operative Day
